# 

**Published:** 1921-07-02

**Authors:** 


### Personalia.

Mr. A. W. Sheen, C.B.E., M.S., M.D., F.R.C.S., has been appointed Professor of Surgery of the University College of South Wales and Monmouthshire. For twenty-seven years on the active staff of the King Edward VII. Hospital, Cardiff, Mr. Sheen conceived and planned medically the Welsh Hospital at Netley. In India a 3,000-bed hospital, complete in all departments, was rapidly produced under his direction. Mr. Sheen's work as consultant in India during the war was recognised by his being invited by the War Office to return as consulting surgeon on the Afghan frontier. His latest appointment was that of visiting surgeon at the Special Surgical Hospital, Shepherd's Bush, and Orpington Hospital of the Ministry of Pensions.

### Home Helps Schemes.

The Health Committee of the Camberwell Borough Council has prepared what it describes as a "workable" scheme for the provision of home helps, and mentions £500 as the cost to the rates for twelve months. The proposal is for the Infant Welfare Centres, Midwives' Associations, and Care Committees to be asked whether they can supply the names of suitable persons as home helps, and also to insert advertisements in the local papers. Persons willing to act as helps should be interviewed by a sub-committee and a register should be kept by the medical officer of health containing the names and addresses of the selected women. The women should be paid at the rate of 1s. 6d. per hour for a forty-seven hour week. All applications for the services of helps should be considered on their merits, and persons provided with helps and who are in a position to make payments should be called upon to do so, the amounts to be fixed by the sub-committee on a sliding scale according to the family circumstances. The authorities at Birmingham appear to have developed a useful corps of home helps. A report of the Health Committee states that the arrangements for the supply of home helps to families during the confinement of the mother are now in operation, and proving to be a great boon. Fifty helps are employed, and a charge of 5s. per day is ordinarily made for their services.

### THE COLLEGE OF NURSING.

**(Limited by Guarantee.)**

### The Annual Meeting and Conference.

Over three hundred members of the College of Nursing attended the Annual Meeting, held on Friday, June 24, in the Surgeon's Hall, Nicholson Street, Edinburgh. In spite of railway difficulties, London, English Centres, and Scottish Centres were well represented. The meeting was presided over by the Hon. Sir Arthur Stanley, G.B.E., C.B., Chairman of the Council.

The adoption of the annual report was moved by Sir Arthur Stanley. Commenting on the attendance, he said the fact that there were such a large number of members present when travelling was so difficult and so expensive was the best proof that they could possibly have that the College was very much alive. One item in the report which he noted with very great satisfaction was the membership. When they founded the College they always took as a goal towards which they had to work a membership of 20,000. This year he was very proud to be able to report that they had over 20,000 nurses as members. A good many had joined since the report was published, and the membership now stood at 20,518 members, all of them fully trained, qualified nurses except a few doctors and a few of those very inferior people called laymen, of whom he was one. The increase in membership during the past year had been just on 3,000. That was a very satisfactory figure, because some 880 of these had joined since not only the entrance-fee of a guinea, but also the annual subscription of 5s. had been decided on. When the founder-members joined the only liability was a guinea down; but owing to the expansion of the work of the College it was found necessary to ask those members if they would, of their own free will and accord, give 5s. yearly. Of the founder-members, 4,994 had agreed to pay 5s. a year, and 276 had paid for a life membership, £5. In other words, 5,000 of their members had voluntarily agreed to give an annual subscription of 5s., which was very encouraging, as it provided an additional income that they could count upon of something over £1,000.

### The Need for Alertness.

The next item in the report of special interest was that the Carnegie Trustees had granted them £500 as a nucleus for a library. This sum would be a very good start to a library which they hoped would be the most complete and perfect library of everything to do with nursing that existed in the world. The College *Bulletin* had met with a very favourable reception, but unfortunately it had involved them in a loss of £590 on the year. This was not really very serious; but a paper like that, with a circulation among over 20,000 members, ought certainly to be able to get enough advertisements to make it pay its way. Then they came to the Unemployment Insurance Act. That was about the only disappointing thing that he had to record this year. They were asked by the Government authorities to find out what the opinion of the nurses was about the Act. From everything that they heard they had reason to believe that the feeling among the nurses was very strongly against coming under that Act. They agreed to have a referendum. He was sorry to say that out of the whole 20,000 members they only got about 5,000 answers. The consequence was that when they went to the Government authorities their case was seriously weakened. It was rather discouraging when they made an attempt like that to get the opinion of the nurses to find that such an enormous number paid no attention whatever to their letters.

### A Superannuation Scheme.

During the year they had also brought up to date and revised the scale of salaries—revising, of course, in these times meant putting up. This scale of salaries was now in force, and it was very satisfactory to know that in a great many hospitals their scale was looked up to as the standard. Everybody wanted to pay the nurses fairly, but unfortunately many hospitals had not the wherewithal. They were working very hard to get the money, and he hoped they would reach the standard of salaries very soon. As to superannuation, the College is having a scheme worked out by some very competent insurance officials, and he hoped before this time next year it would be able to put forward some scheme which would meet with the approval of the nurses and of the hospital authorities.

### Some Good Friends.

Sir Charles Russell had given legal advice during the year to no less than forty-one cases, absolutely free of charge. The College was also fortunate in Sir John and Lady Martin Harvey, who worked so generously for the Home of Rest at Bonchurch, and were working for the College in Canada. Sir Arthur also spoke of the latest extraordinarily generous donation that had been made by Lord and Lady Cowdray, who had presented the College of Nursing with practically a freehold of one of the best houses in London, and had offered to erect a College building on a most suitable site in Henrietta Street. The total value of their gift would not be far short of £100,000.

Turning for a moment to the accounts, the working of the year showed a deficit of £748. A comparatively small deficit like that showed that the College was really working up to its full measure. The Endowment Fund of the College, which was £42,500 last year, had risen to £48,000 this year. The fund for distressed nurses had reached the figure they had aimed at—£100,000.

Miss Hill, R.R.C. (Aberdeen), seconded, and the report was adopted. Miss White moved a vote of thanks to the Council, which Miss Thomas seconded.

### The New Council Members.

The Chairman then read the names of the newly elected Council members—viz.: *For England and Wales,* Viscountess Cowdray, Mr. Comyns Berkeley, Colonel Sir James Cantlie, Miss Amy Hughes, Miss E. C. Barton, Mr. C. E. L. Lyle, M.P., Miss E. G. Love, Miss Margaret Hogg; *for Scotland,* Miss Gill and Dr. John Glaister; *for Ireland,* Sir Andrew Beattie and Professor R. J. Johnston.

On the motion of Miss Lloyd Still, seconded by Miss Gill, Dame Sarah Swift was elected a Vice-President of the College, and the meeting concluded with various votes of thanks.

A Garden Party for the members attending the Conference was held after the meeting in the grounds of the Royal Infirmary, where tea was served.

### Scientific, Social and Economic Questions.

A conference of nurses was held in the evening in the Surgeons' Hall, when Mrs. George Kerr, L.D., J.P., presided over a large attendance. In opening the meeting Mrs. Kerr said that she felt somewhat out of place as the only lay member at the meeting. After having served for more or less twelve years as the Chairman of the Nurses' Committee of Edinburgh Royal Infirmary, however, she sometimes felt that she was almost a nurse. But that was not the case, she found, when she tried to look like a nurse.

### The Nurse's Part in Scientific Medicine.

An address was then given on "The Nurse in Relation to the Development and Practice of Scientific Medicine," by Dr. J. C. Meakins, Professor of Therapeutics at the

(*Continued on p. vi.*)

July 2, 1921] [Price Twopence

### The Hospital

**RATES OF SUBSCRIPTION** (post free, payable in advance).

**Three months, 3/3; six months, 6/6; twelve months, 13/-. (Should the cost of printing and paper as well as increased postage, necessitate alteration of these rates, the period paid for will be reduced proportionately on unexpired subscriptions.) THE HOSPITAL "Index" to Volume LXIX., October 1920 to March 1921, is now ready, price 1/1 per copy, post free.** Address The Manager, The Hospital, "The Hospital" Building, 28/29 Southampton Street, Strand, London, W.C. 2.

